# Effect of NETs/COX-2 pathway on immune microenvironment and metastasis in gastric cancer

**DOI:** 10.3389/fimmu.2023.1177604

**Published:** 2023-04-20

**Authors:** Ange Zhang, Xiaoming Zou, Shifeng Yang, Hao Yang, Zhen Ma, Jiacheng Li

**Affiliations:** ^1^ Department of Gastrointestinal Surgery, The Second Affiliated Hospital of Harbin Medical University, Harbin, Heilongjiang, China; ^2^ The Key Laboratory of Myocardial Ischemia, Ministry of Education, Harbin, China; ^3^ Department of General Surgery, The First Affiliated Hospital of Jiamusi University, Jiamusi, Heilongjiang, China

**Keywords:** neutrophil extracellular traps (NETs), gastric cancer, metastasis, Cyclooxygenase-2 (COX-2), toll-like receptor 2 (TLR 2)

## Abstract

**Background:**

Neutrophil extracellular traps (NETs) are crucial in the progression of several cancers. The formation of NETs is closely related to reactive oxygen species (ROS), and the granule proteins involved in nucleosome depolymerization under the action of ROS together with the loosened DNA compose the basic structure of NETs. This study aims to investigate the specific mechanisms of NETs promoting gastric cancer metastasis in order to perfect the existing immunotherapy strategies.

**Methods:**

In this study, the cells and tumor tissues of gastric cancer were detected by immunological experiments, real-time polymerase chain reaction and cytology experiments. Besides, bioinformatics analysis was used to analyze the correlation between cyclooxygenase-2 (COX-2) and the immune microenvironment of gastric cancer, as well as its effect on immunotherapy.

**Results:**

Examination of clinical specimens showed that NETs were deposited in tumor tissues of patients with gastric cancer and their expression was significantly correlated with tumor staging. Bioinformatics analysis showed that COX-2 was involved in gastric cancer progression and was associated with immune cell infiltration as well as immunotherapy. *In vitro* experiments, we demonstrated that NETs could activate COX-2 through Toll-like receptor 2 (TLR2) and thus enhance the metastatic ability of gastric cancer cells. In addition, in a liver metastasis model of nude mice we also demonstrated the critical role of NETs and COX-2 in the distant metastasis of gastric cancer.

**Conclusion:**

NETs can promote gastric cancer metastasis by initiating COX-2 through TLR2, and COX-2 may become a target for gastric cancer immunotherapy.

## 1 Introduction

The global morbidity and mortality of gastric cancer (GC) are increasing annually, and China has approximately half of the world’s patients with GC (1, 2). Gastric cancer has a complex etiology and is induced mainly by *Helicobacter pylori* infection during an inflammatory reaction (3, 4). The prognosis of GC is polarized. Physical examination shows that endoscopic resection usually leads to a good long-term prognosis of early gastric cancer, but advanced GC is often correlated with distant organ metastasis, leading to poor survival rate and prognosis (5, 6). Therefore, gastric cancer research has gradually focused on the metastasis (7). According to research, GC is always accompanied by the infiltration of a lot of inflammatory cells, which are also implicated in the metastasis of GC (8). Evidence supports that neutrophils play a crucial role in GC (9–11).

Neutrophils are important immune cells in the human body, mainly involved in the inflammatory responses, and the mechanisms of neutrophils in cancer evolution are still unclear (12). Neutrophils promote metastasis of many types of cancers, including gastric cancer (13), which is related to neutrophil extracellular traps (NETs) or their interactions with various inflammatory cytokines (14–17).

NETs are mainly composed of the DNA reticular released by the stimulation and activation of neutrophils, and embedded with various granule proteins, like citrullinated histone H3 (citH3), neutrophil elastase (NE) and myeloperoxidase (MPO) (18). NETs are correlated with the immune response of cancers, like breast cancer, hepatoma, and colorectal carcinoma (19). Our group previous study has reported that NETs can promote gastric cancer metastasis with the underlying mechanisms unclear (20).

As an inducible enzyme, COX-2 can be activated by inflammatory factors and tumor promoters, and its expression is related to the colonization of *Helicobacter pylori*; however, the related mechanisms are unclear (21–23). The research have revealed COX-2 is related to the metastasis of breast, lung, prostate, and ovarian cancers (24). Clinical studies demonstrate that COX-2 can be used as a protein marker for predicting lymph node metastasis of GC (25–27). Neutrophil aggregation and NETs formation can regulate COX-2 in inflammatory diseases like arthritis (28). This study aims to further investigate the mechanisms which NETs promote gastric cancer metastasis and clarify the role of COX-2 in the downstream cascade induced by NETs.

## 2 Materials and methods

### 2.1 Tissue samples and cells

All patients had a clear pathological diagnosis (primary gastric cancer) and signed an informed consent form in this study. We excluded patients under 18 years of age or over 70 years of age, as well as patients with severe cardiovascular and autoimmune diseases. In addition, patients with a prior diagnosis of malignancy or the presence of distant metastases from the tumor were excluded. AGS and HGC-27 were purchased from PROCELL (Wuhan, China). Upon reaching 80-90% cell confluence, follow-up experiments were performed, including cell passaging, cell cryopreservation, and the extraction of RNA and protein.

### 2.2 Neutrophils isolation

We used a neutrophil isolation kit to isolate the neutrophils from peripheral blood of patients with gastric cancer. After mixing the neutrophil separation solution with the blood, the mixture was centrifuged for 35 minutes to stratify the blood, and then the neutrophil layer was gently aspirated with a disposable dropper. The erythrocyte separation solution was used to purify the neutrophil layer. These steps were repeated until red blood cells were completely removed.

### 2.3 Generation, isolation, and preparation of NETs

Using 100nM phorbol 12-myristate 13-acetate (PMA), neutrophils were activated and incubated for four hours. Neutrophils and NETs were collected in cold PBS and centrifuged for ten minutes. To obtain a NETs suspension, the supernatant was centrifuged at 15000g. NETs were frozen at -20°C following the determination of DNA concentration on an ultraviolet spectrophotometer.

### 2.4 Cell stimulation and gene knock-down

To pre-stimulate the cancer cells with NETs, AGS and HGC-27 cells were seeded in a medium containing NETs (0.5μg/ml) for 24 hours. Cancer cells were cocultured with the COX-2 inhibitor NS-398 (50μM, 24 h) (Beyotime, Shanghai, China), and the toll-like receptor 2 (TLR2) inhibitor C29 (50μM, 2h) (MedChemExpress) was pre-stimulated before NETs stimulation. The cells were rinsed with PBS and follow-up experiments were performed after changing the culture medium. The COX-2 knock-down lentiviral vector (sequence: GCTGAATTTAACACCCTCTAT) and negative vector (shNC) (Genechem, Shanghai) were transfected into AGS and HGC-27 cells, and stable cell lines were developed and refrigerated at -80°C.

### 2.5 Real-time polymerase chain reaction (PCR) analysis

RNA was isolated using Trizol reagent (Invitrogen, CA, USA). Applied Biosystems 7500Fast (Thermo Fisher, USA) was used for analysis. Sequences of primers include COX-2, forward:5’-CCAGAGCAGGCAGATGAAATA-3’;COX-2, reverse: 5’-CAGCATCGATGTCACCATAGAG-3’; TLR2, forward:5’-ATCCTCCAATCAGGCTTCTCT-3’;TLR2, reverse:5’-GGACAGGTCAAGGCTTTTTACA-3’; TLR4, forward:5’-AGACCTGTCCCTGAACCCTAT-3’;TLR4, reverse:5’-CGATGGACTTCTAAACCAGCCA-3’; TLR9, forward:5’-AATCCCTCATATCCCTGTCCC-3’;TLR9, reverse:5’-GTTGCCGTCCATGAATAGGAAG-3’.

### 2.6 Transwell assay

Cells were aliquoted into Transwells chambers (Corning, USA), which were inserted in a 24-well plate. After culturing the cells for 24h, the underside of the polycarbonate membranes were fixed. The cells passing through the membrane were observed under a microscope to evaluate the cancer cell migration. In the invasion test, the Matrigel was placed at the bottom of the chamber before cell inoculation. The Matrigel (Corning, 356234, USA) and the 24-well plate were pre-cooled before the Matrigel was laid and then transferred to a 37°C incubator after the Matrigel was laid evenly.

### 2.7 Wound-healing assay

Gastric cancer cells in the pretreated group or untreated control group were counted, and the cell concentration was adjusted. The cells were inoculated evenly in a 6-well plate. When cell convergence reached 80-90%, a wound was made with a 200μL aseptic pipette tip perpendicular to the bottom of the plate.

### 2.8 Immunohistochemistry assay

The sliced tumor tissues were dewaxed with xylene before the experiment. After removing endogenous peroxidases, the tissues were steamed in the citric acid buffer to expose antigen-binding sites. Following blocking with serum, primary antibodies against citH3 (Affinity, USA), COX-2 (Abcam), and TLR2 (Proteintech) were added and incubated overnight. The slides with tissue were plated with a secondary antibody at room temperature, and stained with diaminobenzidine and hematoxylin, which can be preserved for a long time after being treated with neutral gum. Tissues were observed microscopically and analyzed using Image-lab software to evaluate the expression of target proteins.

### 2.9 Immunofluorescence

The tumor cells were seeded on a microscope slide, and the cells were pre-stimulated after completely attached to the slide. For frozen tissue sections, PBS was used to wash off the OCT compound before the experiment. The slides or frozen sections were blocked for one hour, and covered with the primary antibodies against citH3 (Affinity), COX-2 (Abcam), TLR2 (Abcam), and MPO (Abcam) overnight. Cells or tissues were covered with fluorescent secondary antibodies for 2 h, and re-stained with DAPI or Hoechst. We used Image-Lab to evaluate the expression of the target proteins.

### 2.10 Animal model

BALB/c nude mice were purchased from Weitong Lihua Co. Ltd. All mice were anesthetized with inhalation before operating. Intraperitoneal injection of Dnase-1 could inhibit the formation of NETs in nude mice. For the mouse subcutaneous tumor model, 200μL of HGC-27 cell suspension was injected into the axilla, and the mice in experimental group were intraperitoneally injected with deoxyribonuclease I (Dnase-1) every 24h (5mg/kg). All mice were euthanized after 15 days. For the mouse liver metastasis model, 75-125μL of HGC-27 cell suspension was injected into the spleen. After ligating the blood vessels around the spleen, the spleen was severed. Tumor tissues were collected for follow-up experiments.

### 2.11 Data preparation and processing

Disease expression data and clinical information of gastric cancer (STAD) were obtained from the Cancer Genome Atlas (TCGA) database. The transcriptional spectra of tumor and paracancerous samples were obtained from the TCGA. The response of STAD to immunotherapy was evaluated by a submap analysis. The database of GDSC was used to forecast the drug sensitivity.

### 2.12 Carcinogenic characteristics of COX-2

The expression differences of the target gene in tumor and adjacent tissue were analyzed based on transcriptional data, and the Kaplan-Meier curve was plotted based on clinical information. GO and KEGG databases were used to analyze the correlation between COX-2 and cancer-related functional pathways, and the results were displayed by GSVA thermomaps.

### 2.13 The immunological characteristics associated with COX-2

We employed ESTIMATE algorithm to calculate the infiltration ratio. The relationship between neutrophil infiltration and the target gene level was assessed using the MCPcounter algorithm with the Tumor Immunoassay database (TIMER 2.0), and ssGSEA was performed with the GSVA program package.

### 2.14 Statistical analysis

The independent samples with normal distribution were tested using the parametric t-test, the samples with homogeneity of variance were tested using the nonparametric t-test, and the samples with uneven variance were tested using Welch’s correction. The paired samples whose difference obeyed a normal distribution were tested using a parameter t-test. Paired samples whose difference did not obey a normal distribution were analyzed using a nonparametric t-test. Statistical significance was set at p< 0.05.

## 3 Results

### 3.1 NETs and COX-2 in gastric tumor tissues

**Figure 1:**
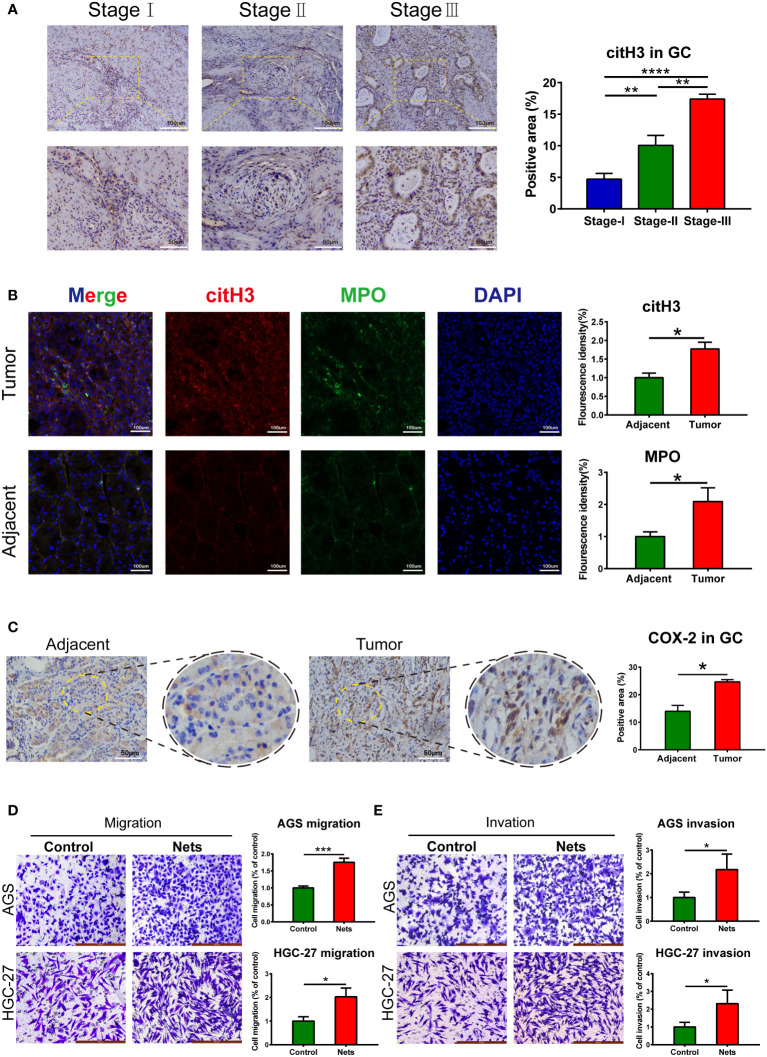
NETs and COX-2 in gastric tumor tissues. **(A)** Expression of cit-H3 in gastric cancer tissues of different stages. (**p<0.01; ****p<0.0001; n=3) **(B)** Expressions of cit-H3 and MPO separately in gastric cancer and adjacent tissues. The expression of both was assessed by fluorescence intensity. (*p<0.05 vs. Adjacent; n=3) **(C)** Representative images of COX-2 expression in gastric cancer and adjacent tissues. The expression of COX-2 was compared by statistical immunohistochemical positive rate. (*p<0.05 vs. Adjacent; n=3) **(D, E)** The cells crossing the polycarbonate membrane in the control and NETs stimulation groups were measured by migration and invasion assays. (*p<0.05 vs. Control; n=3). All results are presented as mean ± SD.

The markers of NETs, citH3 and MPO, were detected in GC. In the frozen sections, the MPO (green fluorescence) and citH3 (red fluorescence) were full of disordered tumor tissues (**Figure 1B**), and their fluorescence intensities were higher than those of normal tissues adjacent to tumors. Similarly, immunohistochemistry demonstrated the citH3 in GC was correlated with the pathological stage (**Figure 1A**). These observations proved the high expression of NETs in GC. We then examined the COX-2 level in tumor tissues. The immunohistochemistry indicated COX-2 in tumor tissues was considerably increased than that in adjacent (**Figure 1C**). To investigate the effect of NETs on the invasion and migration ability, we also employed Transwell assay, the research indicated the number of cells which were pre-stimulated by NETs moving through the membrane was higher than that of the untreated gastric cancer cells (**Figure 1D**). Similarly, after covering the polycarbonate membrane with Matrigel, the number of cells pre-stimulated by NETs was still higher than that of the untreated cells (**Figure 1E**).

### 3.2 COX-2 is correlated with prognosis and involves in the progression of GC

**Figure 2:**
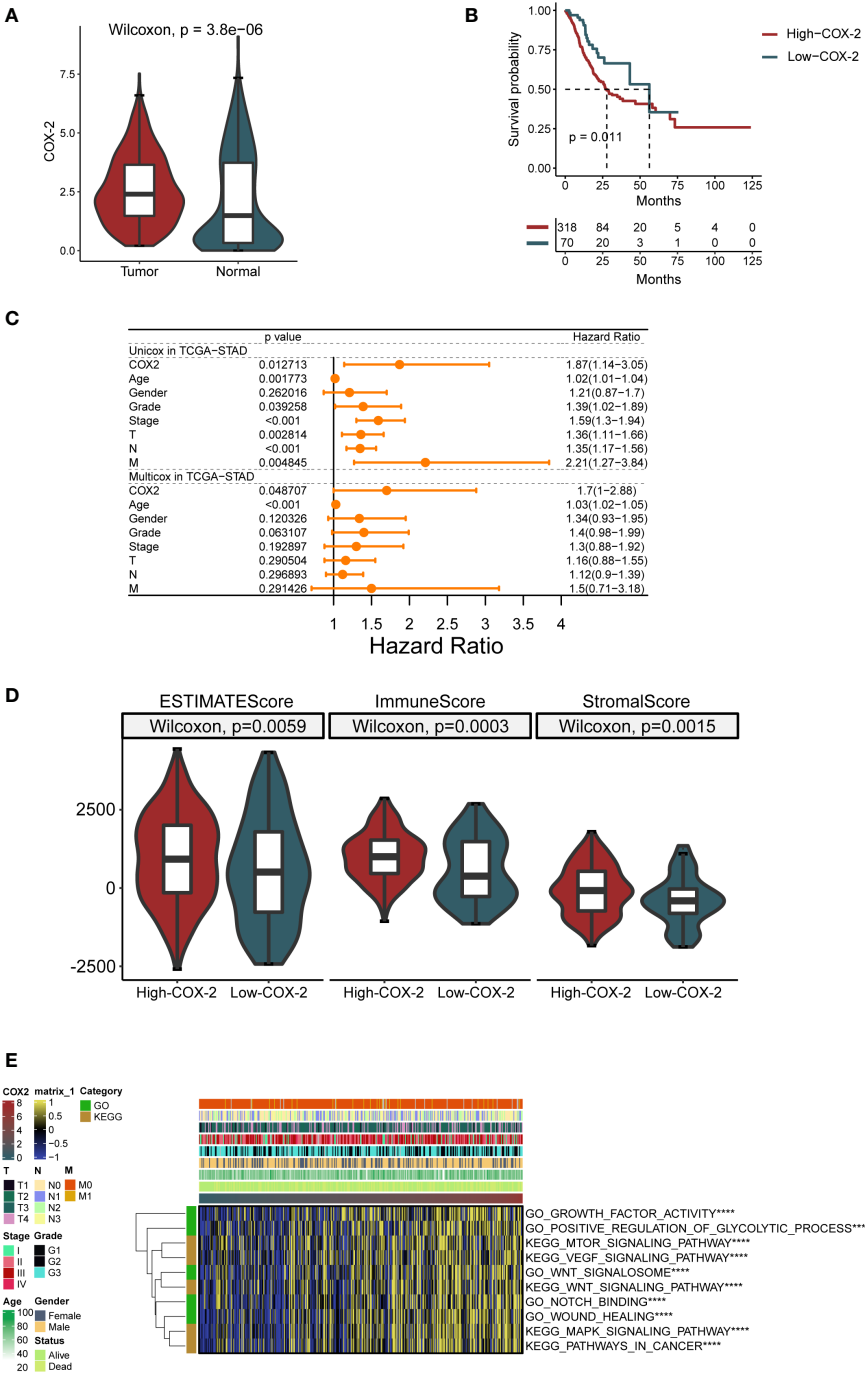
COX-2 is correlated with GC progression and prognosis. **(A)** Difference of COX-2 expression between cancer and paracancerous samples in TCGA database. **(B)** The survival curve of patients with high and low COX-2 expression based on TCGA database. **(C)** Forest map of univariate and multivariate cox regression based on TCGA dataset and clinical variables. **(D)** In gastric cancer tumor microenvironment, the expression of COX-2 is related to ESTIMATE score, immune cell infiltration (ImmuneScore) and the proportion of stromal cells (StromalScore). **(E)** The GSVA analysis of tumor-associated functions and signaling pathways in GO and KEGG databases.

The analysis based on the TCGA database showed that COX-2 was highly expressed in tumor tissues of GC (**Figure 2A**). Furthermore, The Kaplan-Meier curve depicts the change in survival probability over time in high and low COX-2 groups (**Figure 2B**). The gene expression and clinical data in TCGA database were analyzed by univariate and multivariate cox regression analysis, and the result proved that COX-2 was an independent risk factor in GC (**Figure 2C**). The TME score revealed that COX-2 level was positively correlated with immune score and stromal score, which implied that the activation of COX-2 was involved in the change of TME in GC (**Figure 2D**). Through Gene Set Variation Analysis (GSVA) of tumor-associated functions and signaling pathways in GO and KEGG databases, we found that COX-2 was correlated with tumor progression. The results of GSVA indicated that high COX-2 level was related with biological processes which contribute tumor advance, such as growth factor activity and positive regulation of glycolysis, and it was also associated with a variety of signaling pathways which accelerate tumor development, such as VEGF and MAPK signaling pathways (**Figure 2E**).

### 3.3 COX-2 in GC is related with immunocyte infiltration and immunotherapy

**Figure 3:**
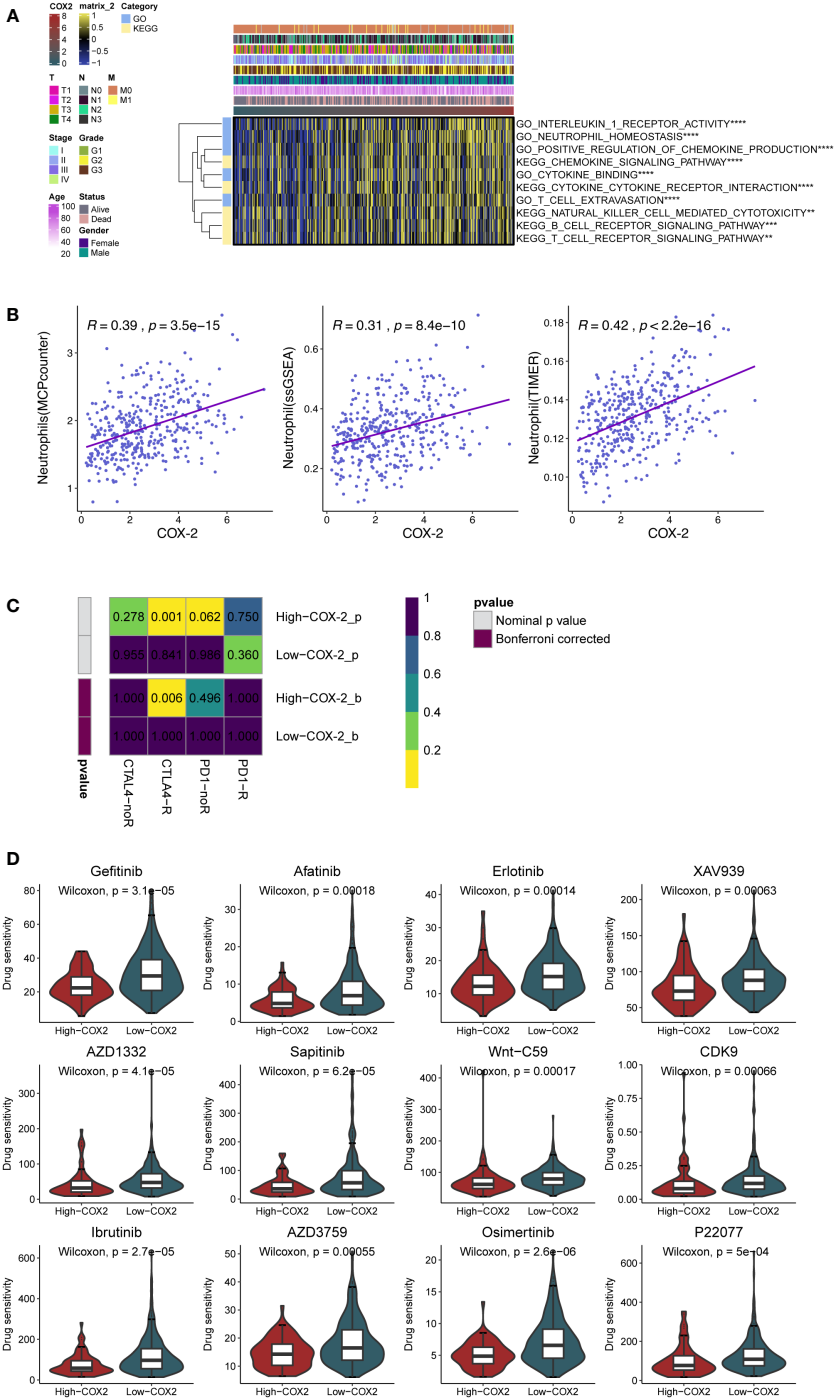
COX-2 is associated with immunocyte infiltration and immunotherapy. **(A)** The GSVA analysis of the processes related to the immune reaction. **(B)** The relationship between the expression of COX-2 and neutrophil infiltration in gastric cancer was analyzed by MCPcounter algorithm, single sample genome enrichment analysis (ssGSEA) and TIMER database. **(C)** The submap analysis between immunotherapy responses (anti-PD-1 and anti-CTLA-4) and COX-2 levels in TCGA-STAD. **(D)** The evaluation of drug sensitivity in different COX-2 levels based on GDSC database.

We employed GSVA to analyze the processes related to the immune reaction, such as the signaling pathways relevant to T/B cells and cytokines. The result demonstrated that the COX-2 level was closely linked to the immune response and immunocyte infiltration in GC (**Figure 3A**). Subsequently, we separately evaluated the neutrophils in GC by multiple analysis methods. The results indicated that COX-2 was significantly correlated with neutrophil deposition (**Figure 3B**). Immune checkpoint inhibitors are the most established and widely used in the immunotherapy of tumors. Therefore, we performed submap analysis on the correlation between COX-2 and the immune checkpoint inhibitors response. The results showed that a high COX-2 level was correlated with anti-CTLA4 response (**Figure 3C**). In addition, we also used the GDSC database to analyze the sensitivity of chemotherapeutic drugs at different COX-2 levels. We found the high COX-2 group in GC presented lower drug sensitivity to Gefitinib, Afatinib, Erlotinib, XAV939, AZD1332, Sapitinib, Wnt-C59, CDK9, Ibrutinib, AZD3795, Osimertinib and P22077 (**Figure 3D**).

### 3.4 NETs promote gastric cancer metastasis by regulating COX-2

**Figure 4:**
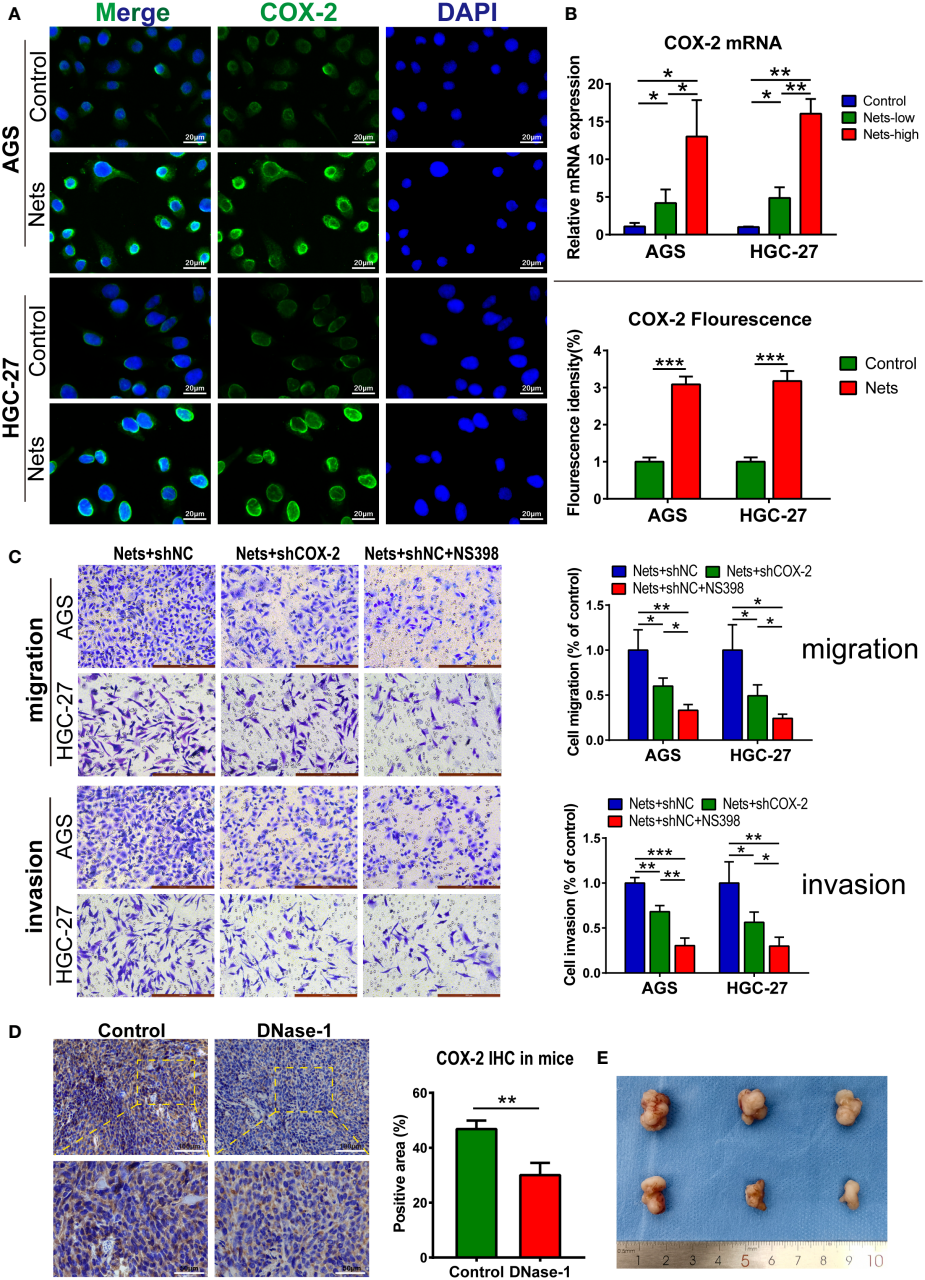
NETs promote gastric cancer metastasis by regulating COX-2. **(A)** Representative images of COX-2 distribution and expression in two gastric cancer cell lines after NETs stimulation. The COX-2 expression was evaluated by statistical fluorescence intensity. (***p<0.001 vs. Control; n=3). **(B)** The mRNA expression of COX-2 in two gastric cancer cell lines changed with the NETs concentration. (*p<0.05; **p<0.01; n=3) **(C)** The number of cells stimulated by NETs passing through Transwell chambers was measured by migration and invasion assays and observed after crystal violet staining. NS398 addition and shCOX-2 transfection were used to simulate the inhibition of COX-2 in different degrees. Each group maintained the same concentration of NETs. (*p<0.05; **p<0.01; ***p<0.001; n=3) **(D, E)** The expression of COX-2 in subcutaneous tumor was decreased after the nude mice were intraperitoneal injected Dnase-1. We use the positive rate of immunohistochemistry to measure the expression of COX-2. Subcutaneous tumor specimens are shown on **(E)**. (**p<0.01 vs. Control; n=3). All results are presented as mean ± SD.

We measured COX-2 expression in low- and high-concentration NETs stimulation (0.25 and 0.5μg/ml) and compared it with the control group to explore the relationship between NETs and COX-2. Compared with cells not stimulated by NETs, the COX-2 mRNA expression was increased after the pre-stimulation of NETs-conditioned medium, and the COX-2 mRNA was correlated with the NETs concentration positively (**Figure 4B**). To observe COX-2 expression in cells stimulated by NETs, we performed immunofluorescence analysis, and the fluorescence intensity of COX-2 was significantly increased when cells were pretreated with NETs. We observed that COX-2 (green fluorescence) was mainly located in the nuclear membrane of AGS cells, and was pervasively expressed in the cytoplasm. Compared to the dim green fluorescence of the control group, the fluorescence intensity of COX-2 in AGS cells pretreated with NETs was significantly increased, suggesting that NETs stimulation regulates the COX-2 expression, similar to that in HGC-27 cells (**Figure 4A**). We employed Transwell assay to research the function of COX-2 in cells stimulated by NETs. We used cells stimulated by NETs alone as the control group, the gastric cancer cells without COX-2 knockdown (shCOX-2) were transfected with the shNC, and the control group maintained the same concentration of NETs stimulation as the experimental groups. In the experimental group of cells transfected with shCOX-2, the amount of cells moving through the polycarbonate membrane reduced after NETs stimulation. In the other experimental group cocultured with selective COX-2 inhibitors (NS398), the amount of cells moving through the polycarbonate membrane further reduced. After the polycarbonate membrane was covered with Matrigel, the results of the invasion experiment were consistent with the above migration assay (**Figure 4C**). To verify the correlation between NETs stimulation and COX-2, we then employed experiments *in vivo*. We subcutaneously inoculated HGC-27 cells to nude mice under the same conditions, and injected Dnase-1 into the experimental group of animals to inhibit NETs. Compared with the control, the COX-2 in experimental group significantly reduced following the treatment with Dnase-1 (**Figures 4D, E**).

### 3.5 TLR2 is the pivot for NETs to regulate COX-2

**Figure 5:**
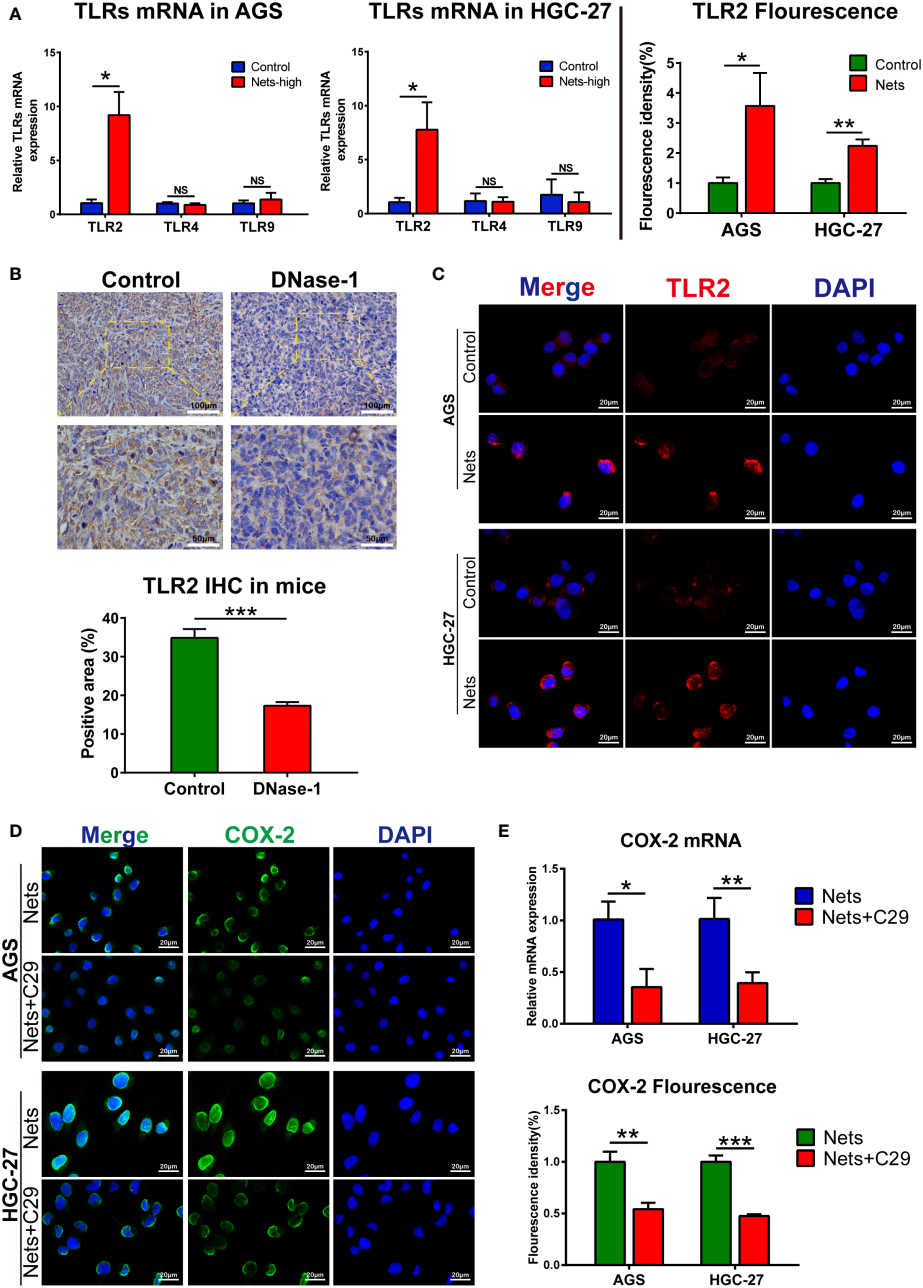
TLR2 is the pivot for NETs to regulate COX-2 **(A)** The mRNA expression of TLRs in two gastric cancer cell lines after NETs stimulation. (*p<0.05 vs. Control; NS, no significance; n=3) **(B)** The expression of TLR2 in subcutaneous tumors was decreased after the nude mice were intraperitoneal injected Dnase-1. (***p<0.001 vs. Control; n=3) **(C)** Representative images of TLR2 distribution and expression in two gastric cancer cell lines after NETs stimulation. The expression of TLR2 was evaluated by statistical fluorescence intensity. (*p<0.05; **p<0.01; n=3) **(D)** TLR2 inhibitor C29 affects the expression of COX-2 in gastric cancer cells stimulated by NETs, the distribution and expression of COX-2 are illustrated in the figure. The expression of target gene was evaluated by its fluorescence intensity. (**p<0.01; ***p<0.001; n=3) **(E)** TLR2 inhibitor (C29) affects the mRNA expression of COX-2 in gastric cancer cells stimulated by NETs, which were measured by real-time PCR. (*p<0.05; **p<0.01; n=3). All results are presented as mean ± SD.

TLR/MyD88-related pathways widely exist in many cancers, like liver cancer (29) and colon cancer (30, 31), regulating the tumor inflammatory microenvironment and development. Previous studies on intestinal tumors (32) and gastric cancer (33) showed that TLR/MyD88 signaling could regulate its downstream COX-2/PGE2 axis. To detect the upstream pathway in which COX-2 expression is regulated by NETS, we screened TLR signals to determine the target of NETs regulation. Studies have shown that TLR2, 4 and 9 are the three most important receptors that regulate the progression of various cancers among the TLR receptors (TLR1-10). TLR2/4 are dominant in gastrointestinal cancers, like esophageal cancer, GC, and colon cancer, while TLR9 signaling is more common in other kinds of cancers, like breast cancer, prostate cancer, and renal cell carcinoma (34). Therefore, we screened TLR2, 4 and 9 after the cells were pretreated with NETs. The experimental group of cells was pre-stimulated by co-culture with NETs in the medium, and then TLR2/4/9 expression were measured compared with the control. After NETs stimulation, the Ct value of TLR4/9 did not change significantly, but the Ct value of TLR2 decreased markedly, indicating that NETs stimulation up-regulated TLR2 expression (**Figure 5A**). To observe TLR2 expression stimulated by NETs, we performed immunofluorescence analysis. In AGS cells, TLR2 (red fluorescence) was located on the cell membrane, and its fluorescence intensity was increased in cells pre-stimulated with NETs. The same phenomena were observed in HGC-27 cells (**Figure 5C**). We then measured TLR2 expression *in vivo*. NETs in tumor tissues was inhibited by Dnase-1 intraperitoneal injection during the tumor formation of HGC-27 cells in mice and compared with the untreated control group. In the experimental group in which NETs were inhibited, the expression of TLR2 was significantly decreased. **Figure 5B** showed the immunohistochemistry results. We have previously shown that COX-2 and TLR2 are affected by NETs stimulation, but the specific relationship between them needs to be further confirmed. The immunofluorescence result indicated COX-2 was decreased by TLR2 inhibitor (C29) in tumor cells treated with NETs. Compared with the control stimulated with NETs alone, the fluorescence intensity of COX-2 in cells pretreated with C29 significantly decreased after NETs stimulation (**Figure 5D**). As determined by PCR, the COX-2 mRNA in C29 treatment group was more decreased than that in group stimulated with NETs only (**Figure 5E**). These results interpret the connection between TLR2 and COX-2 downstream of NETs stimulation.

### 3.6 NETs promote GC metastasis by acting on COX-2 through TLR2

**Figure 6:**
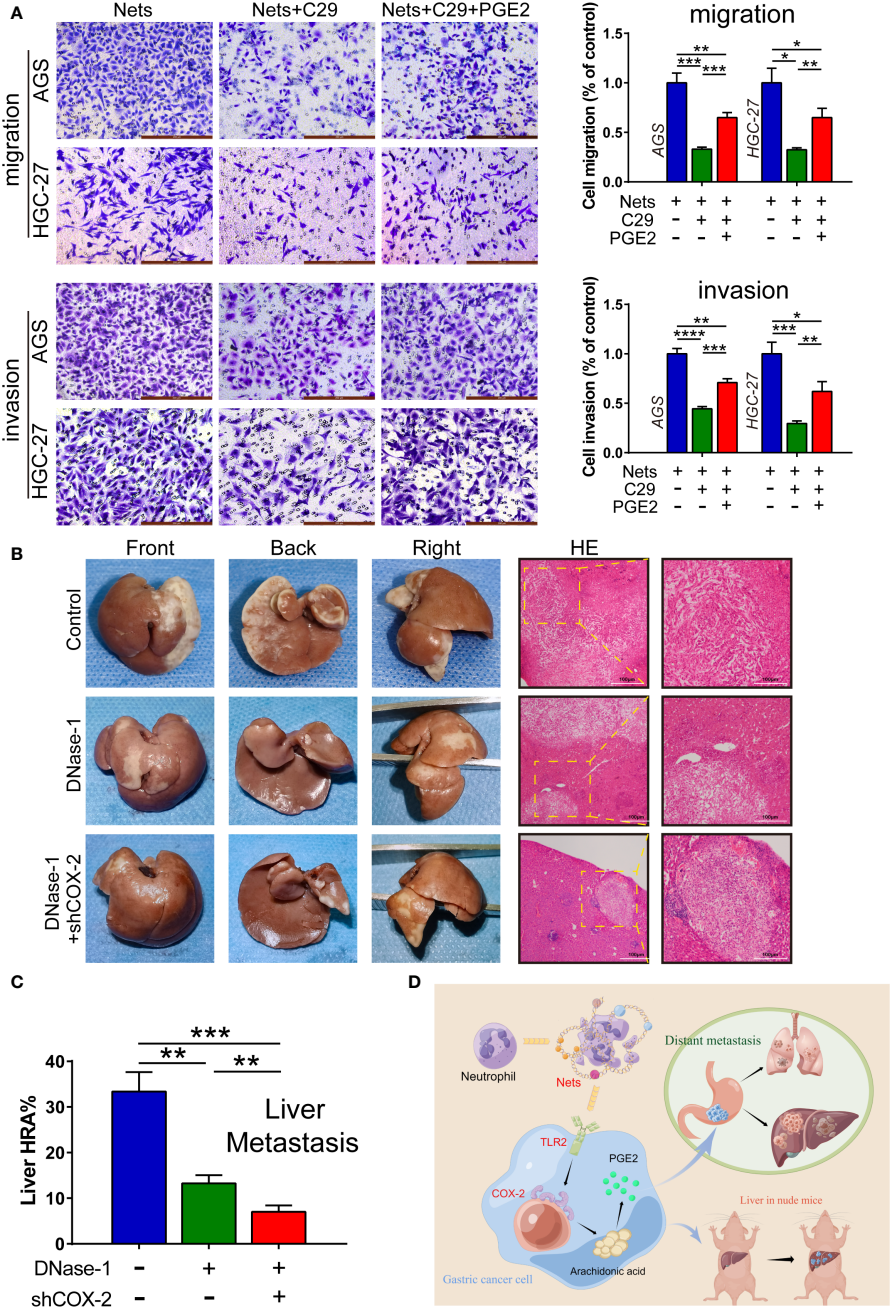
NETs act on COX-2 via TLR2 to promote GC metastasis. **(A)** The number of cells affected by NETs passing through membrane was measured by migration and invasion assays with the intervention of C29 and PGE2, which were observed after staining. (*p<0.05; **p<0.01; ***p<0.001; ****p<0.0001; n=3) **(B, C)** Dnase-1 was injected into mice to inhibit NETs. HGC-27 cells transfected with shCOX-2 or negative control shNC were implanted in the spleen of mice. The proportion of liver tissue replaced by hepatic metastatic tumor was counted as the liver replacement area (HRA %). (**p<0.01; ***p<0.001; n=3) **(D)** The illustration of NETs promoting gastric cancer metastasis by regulating COX-2. (This picture is authorized by the Figdraw platform. ID: SUWSO44938). All results are presented as mean ± SD.

As the main function of COX-2 is to convert arachidonic acid into prostaglandin E2 (PGE2), PGE2 can be measured to quantify the content of COX-2 under the same conditions (35). To further prove the function of NETs in regulating COX-2 through TLR2 in gastric cancer, we used PGE2 to perform rescue experiments. We employed Transwell assay to detect the metastasis potential of cells in the control group and each experimental group stimulated by NETs. As the control group, we employed gastric cancer cells that had only been activated by NETs, whereas the C29-pretreated cells were used as the experimental group 1. PGE2 was added to gastric cancer cells after TLR2 inhibition to simulate COX-2 up-regulation, called experimental group 2. In the migration assay, the amount of migrated cells in experimental group pretreated with C29 before NETs stimulation decreased, but after the addition of PGE2, the amount increased. The invasion assay showed a similar trend (**Figure 6A**). We then employed a liver metastasis model in nude mice. **Figure 6B** shows the multifaceted view of nude mouse liver and HE staining of metastatic tumor sections. We used the hepatic replacement area (HRA) to assess the severity of liver metastasis. The results showed that the inhibition of NETs with Dnase-1 during liver metastasis formation could lead to a significant decrease in HRA compared to the control. After the COX-2 knockdown in HGC-27 cells, we discovered the HRA was further reduced (**Figure 6C**). These results suggest a NETs/TLR2/COX-2/PGE2 pathway in GC, through which NETs promote metastasis.

## 4 Discussion

Neutrophils contribute to the innate immunity of the human body. Their main role is to respond to the recruitment of the inflammatory chemokine and then engulf pathogenic microorganisms in the infected area of the human body (36–39). In the tumor microenvironment (TME), neutrophils support tumor progression primarily through their pro-inflammatory effects, and they also interact with other immune cells. Studies showed that neutrophils cause the immune escape by inhibiting the T cells production (40), leading to tumor progression by affecting the recruitment of tumor-associated macrophages (TAMs) (41). During cancer progression, tumor cells enter the peripheral blood circulation and result in colonization and metastasis in various organs throughout the body. Metastasis is often accompanied by inflammatory reactions (42–44). In this progression, the neutrophils secretion plays a crucial part, promoting angiogenesis factors secretion and extracellular matrix (ECM) degradation which lead to tumor spread (45–48). Additionally, NETs has been proved to facilitate tumor metastasis in many cancers, whereas the underlying mechanisms are still unclear (49–53).

NETs formation is mainly dependent on ROS produced by NADPH oxidase. NETs use citH3, NE, MPO as their markers (18). We found the up-regulation of NETs in gastric tumor by measuring the expression of citH3 and MPO, and these two markers demonstrated the basic morphology and localization of NETs in frozen tumor sections in immunofluorescence analysis (**Figure 1B**). Previous researches demonstrate NETs exist in the extra-tumor matrix (ECM) of gastric tumors, while the main mechanism which promotes metastasis is precisely the remodeling of ECM (54). We found that the metastatic potential of gastric cancer cells was enhanced after the stimulation of NETs by Transwell assay (**Figure 1D**). The researches have demonstrated that both normal human endothelial cells and tumor epithelial cells treated with NETs showed mesenchymal changes (55–57), which indicates that NETs may take part in the metastasis of GC completely, including the proliferation of endothelial cells and the distant spread of tumor cells. This was also verified in an animal model in this study. After intraperitoneal injection of Dnase-1 in mice, the hepatic replacement area (HRA) decreased significantly, indicating that NETs inhibition delayed the metastasis in some extent (**Figure 6B**). Previous studies show that NETs plays a supporting part in the progression of GC, including that NETs promote the formation of a hypercoagulable state, and NETs are also found in peritoneal metastases; however, the mechanism by which NETs affect GC is still unclear (52, 58, 59). With further research, NETs have been shown to induce many pro-inflammatory factors in tumor microenvironment, like IL-8, TNF, and PDL1, during the regulation of cancer. The interaction between these factors and NETs also plays a crucial part in tumor metastasis (60, 61). Similarly, inflammatory factors are significant in development of GC. Inflammatory factors, like COX-2 and IL-6, jointly form the inflammatory network in gastric cancer (62, 63), and the function of COX-2 in colon tumor metastasis has been confirmed *in vivo* (64). Thus far, we have found an interesting phenomenon: the mode which NETs regulate tumor development overlaps with the function of inflammatory factors in gastric cancer, which raises the question about whether NETs can affect tumor progression by regulating these inflammatory factors.

As a member of the tumor inflammatory microenvironment, COX-2 is localized to the nuclear membrane and endoplasmic reticulum (65), and it plays a critical part in many cancers (66–69). For example, COX-2 can regulate intestinal cell adhesion and up-regulate the activity of matrix metalloproteinase to enhance metastasis (70, 71). In addition, the overexpression of COX-2 enhanced the chemotaxis of breast cancer cells to IL-11, thus up-regulating the bone metastasis of tumor (72). In colorectal cancer, adding COX-2 inhibitor to the tumor perioperative combination regimen can inhibit liver metastasis of mice (73). However, the research on COX-2 and inflammatory cells in tumor process has mainly focused on tumor-associated macrophages (TAMs). Researches have demonstrated that M2 TAMs can advance tumor angiogenesis and invasion by regulating COX-2 and up-regulating the matrix metalloproteinase, prostaglandin E receptor (EP1), and other oncogenes (74–76); however, there are few studies on their relationship with neutrophils. Additionally, as a pro-inflammatory factor, COX-2 expresses both in tumor and stromal cells, but the tumor-promoting effect of COX-2 are mainly in tumor (77, 78). We detected that stimulation with different concentrations of NETs could regulate the level of COX-2 in AGS and HGC-27 cells (**Figure 4B**). Bioinformatics analysis revealed a crucial role of COX-2 in TME (**Figures 2D, E**), and its level was closely associated with the deposition of neutrophils (**Figure 3B**). In addition, ROS not only promotes the production of NETs, but also has been shown to up-regulate COX-2 in inflammatory environment (79, 80). Therefore, we speculate that NETs can affect tumor progression through COX-2. Previous researches showed that COX-2 was regulated by various inflammatory mediators to promote tumor cells metastasis (81–84). We observed the effects of COX-2 restraint on the invasion and migration abilities of cancer cells in the NETs environment (**Figure 4C**). The half-inhibitory concentration (IC50) of NS398 was 1.77μmol/L, so the 50μmol/L concentration of NS398 was able to inhibit COX-2 protein more completely. However, the gene knock-down technique was limited by the transfection efficiency, and its inhibitory effect on COX-2 was reduced compared with NS398. The results indicate NETs enhances the metastatic potential of cells by regulating COX-2. In the following study, the liver HRA was decreased compared to the control after the knockdown of COX-2 in HGC-27 cells (**Figure 6B**). The finding may provide a new option for the therapy and prophylaxis of distant metastases in patients with advanced GC. We also found that COX-2 was correlated with the sensitivity to immunotherapy and chemotherapeutic agents, which may provide guidance for medical treatment of GC (**Figures 3C, D**). For patients with high COX-2 expression, the combination of COX-2 inhibitors and immune checkpoint inhibitors may be an effective regimen.

TLRs are a class of transmembrane proteins located on the cell membrane whose main function is to participate in the body’s natural immunity. They are usually located on the surface of dendritic cells and macrophages, but there are few studies on the relationship between TLRs and NETs (85–87). Although most TLRs are expressed in the cell membrane, the localization of some TLRs is altered when normal epithelial cells are transformed into malignant tumor cells, and they translocate from the cell membrane into the cytoplasm (88). The inflammatory response is crucial for tumor development and TLRs associate microbes with inflammatory factors. It has been shown that in colitis-associated colon cancer, inhibition of TLR-related signaling pathways suppresses the inflammatory response and tumor progression (30, 31). Similarly, in the inflammatory microenvironment of gastric cancer, *Helicobacter pylori* activates downstream inflammatory factors, like IL-6, IL-10, and COX-2, through TLRs (mainly TLR2 and TLR4), thus initiating a series of inflammatory responses (33, 89, 90). Since we previously demonstrated the connection between NETs and COX-2 in GC, we questioned whether NETs could regulate COX-2 through TLRs, like *Helicobacter pylori*. We measured the mRNA expression of three TLRs in AGS and HGC-27 cells stimulated by NETs using real-time PCR (**Figure 5A**). The result demonstrated a correlation between TLR2 and NETs. In subsequent experiments, we inhibited TLR2 and introduced PGE2 to the NETs-stimulated environment. The Transwell assay indicated that the metastatic potential of gastric cancer cells was initially suppressed and then promoted, highlighting that TLR2 is essential to the mechanism by which NETs regulates COX-2 (**Figure 6A**). Studies in hepatocellular carcinoma showed that TLR2 was involved in the immune escape initiated by HMGB1 and induced the senescence and autophagy of hepatocytes (91, 92). Furthermore, TLR2 is highly expressed in breast cancer stem cells, and TLR2 inhibition significantly attenuated the lung metastasis in animal models (93). Besides, TLR2 is also a treatment target of melanoma metastasis (94, 95). In addition to the infection by *Helicobacter pylori*, various TLR2 ligands are expressed in GC like HMGB1 and IL-11 (33, 96). IL-11 promoted tumor progression by initiating gp130/Stat3 pathway through TLR2, and cancer metastasis was inhibited by blocking the TLR2 signal in mice (97). Similar to previous studies, our study also showed that TLR2 is involved in tumor progression in gastric cancer, but we demonstrated that NETs could also act as a ligand of TLR2 to initiate downstream inflammatory factors.

In general, our results provide a multi-molecular mechanism by which NETs promote gastric cancer metastasis, emphasize the important role of NETs and COX-2, moreover provide potential targets (NETs, TLR2, COX-2) for the clinical therapy and prophylaxis of the metastasis (**Figure 6D**). Since NETs is a reticular structure composed of many granule proteins, the specific mechanism of NETs promoting metastasis, such as the binding sites of NETs and the function of various granule proteins, remains to be further explored. Furthermore, some studies reported that COX-2-PGE2 has a positive feedback mechanism in tumors and mediates immunosuppression of cancer (98). A similar mechanism may exist in the process of NETs regulation of metastasis, which will be our follow-up research.

## Data availability statement

The original contributions presented in the study are included in the article/supplementary material. Further inquiries can be directed to the corresponding author.

## Ethics statement

The studies involving human participants were reviewed and approved by Internal Audit and Ethics Committee of the Second Affiliated Hospital of Harbin Medical University. Approval number of ethical review: KY2021-075. The patients/participants provided their written informed consent to participate in this study. The animal study was reviewed and approved by Medical Ethics Committee of the second affiliated Hospital of Harbin Medical University. Approval documents for ethical review of animal experiments. Approval number of ethical review: SYDW2021-072.

## Author contributions

AZ, XZ, and SY designed the research, completed the experiments; HY, JL, and ZM took part in the animal experiments. All authors contributed to the article and approved the submitted version.
